# Traumatic Right Atrium Perforation Causing a Pneumothorax and Pneumopericardium, Treated Conservatively

**DOI:** 10.7759/cureus.54566

**Published:** 2024-02-20

**Authors:** Diva Maraj, Omair Ahmed, Muhammad Qureshi, Hussein Othman

**Affiliations:** 1 Internal Medicine, Henry Ford Jackson Hospital, Jackson, USA; 2 Cardiology, Henry Ford Jackson Hospital, Jackson, USA

**Keywords:** conservative, pacemaker lead perforation, pacemaker, pneumopericardium, pneumothorax (ptx)

## Abstract

Pacemaker insertion is a daily occurrence in the United States of America, and it is a relatively common procedure; however, complications can occur. One common complication includes the development of a pneumothorax; however, there are rare instances where patients can develop a pneumopericardium as well. We present a case of a patient who underwent dual chamber pacemaker implantation complicated by a pneumothorax and left-sided pneumopericardium, which is a rare finding. This patient initially presented with syncopal episodes and a dual chamber pacemaker was inserted; however, not long after, the patient developed pericarditis and was found to have a pneumothorax and a pneumopericardium. In these cases, patients can be treated with chest tube insertion, lead extraction, or even conservatively, depending on the patient's clinical status. Various reasons exist for the development of a pneumothorax and pneumopericardium; however, the guidelines on management are still unclear and require further study. In our patient, his pneumothorax and contralateral pneumopericardium were treated conservatively with stable follow-up post-hospitalization.

## Introduction

Around 190,000 patients undergo pacemaker implantation yearly in the United States and although it’s a relatively safe procedure, there can be complications, such as pneumothorax, cardiac tamponade, and infection [1]. Pneumopericardium after pacemaker implantation is an extremely rare complication, as the most common complication after pacemaker implantation is usually a unilateral pneumothorax, with a frequency of 1-2% [1]. A pneumothorax can present with symptoms such as pleuritic chest pain, shortness of breath, or be completely asymptomatic [1,2]. Interestingly, pneumothoraxes are usually always ipsilateral to the pacemaker and contralateral events are rare [1-3]. An even rarer complication includes the development of a pneumopericardium, in which patients may require a chest tube, or even lead extraction [4]. There are currently three other known case reports in the current literature, in which a patient presented with a contralateral pneumopericardium and pneumothorax after pacemaker insertion, which requires the need for further management and research into this condition. We present a case of a 68-year-old male who underwent dual chamber pacemaker implantation complicated by a contralateral pneumopericardium and pneumothorax. This article was previously presented as a meeting abstract at the 2023 American Heart Association Annual Scientific Meeting on November 11th, 2023.

## Case presentation

**Figure 1:**
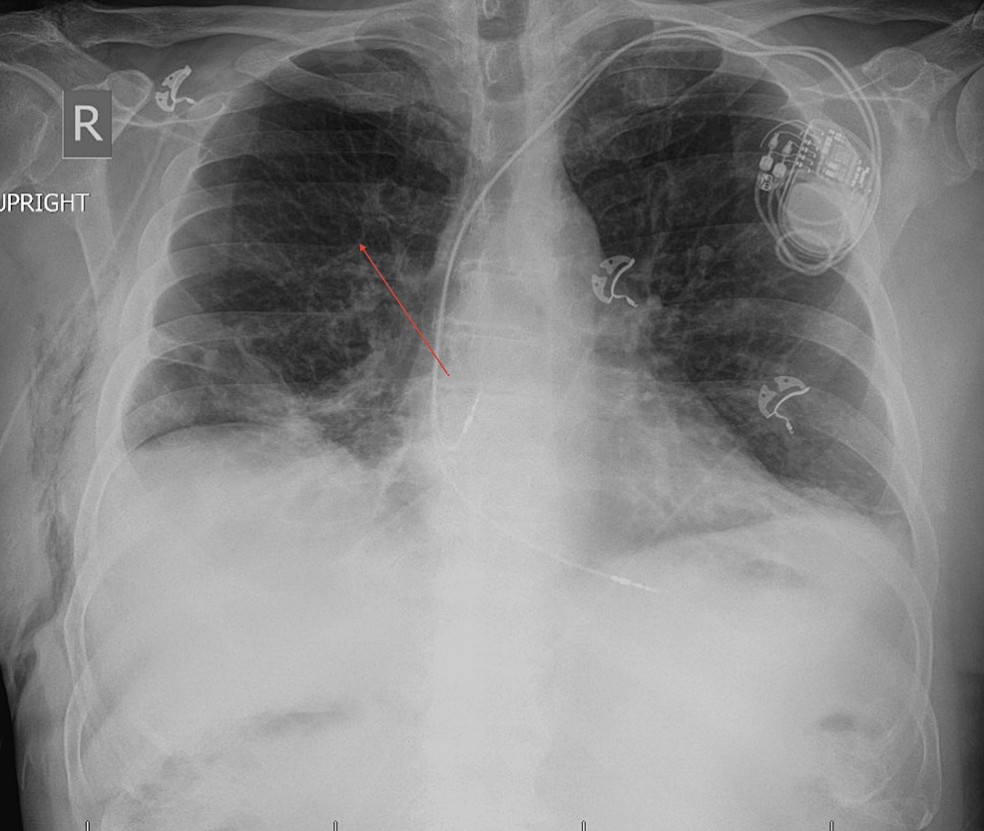
Chest X-ray demonstrating contralateral pneumothorax to the pacemaker, as indicated by the arrow

**Figure 2:**
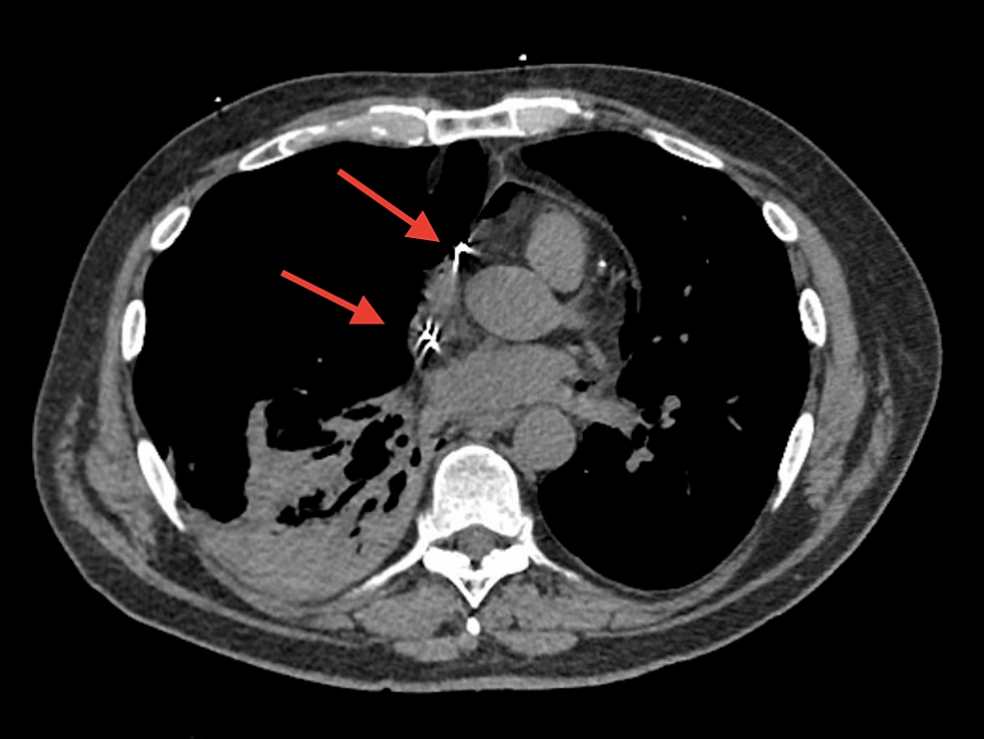
CT of the chest demonstrating tip of wires (red arrows) appears to be in the region of the right atrium, right ventricle region though the right ventricular lead appears to extend beyond the wall of the right ventricle area and it is hard to determine if it is within the wall or within pericardium. Similarly, right atrial lead also appears intended right atrium and perforation is also a possibility.

**Figure 3:**
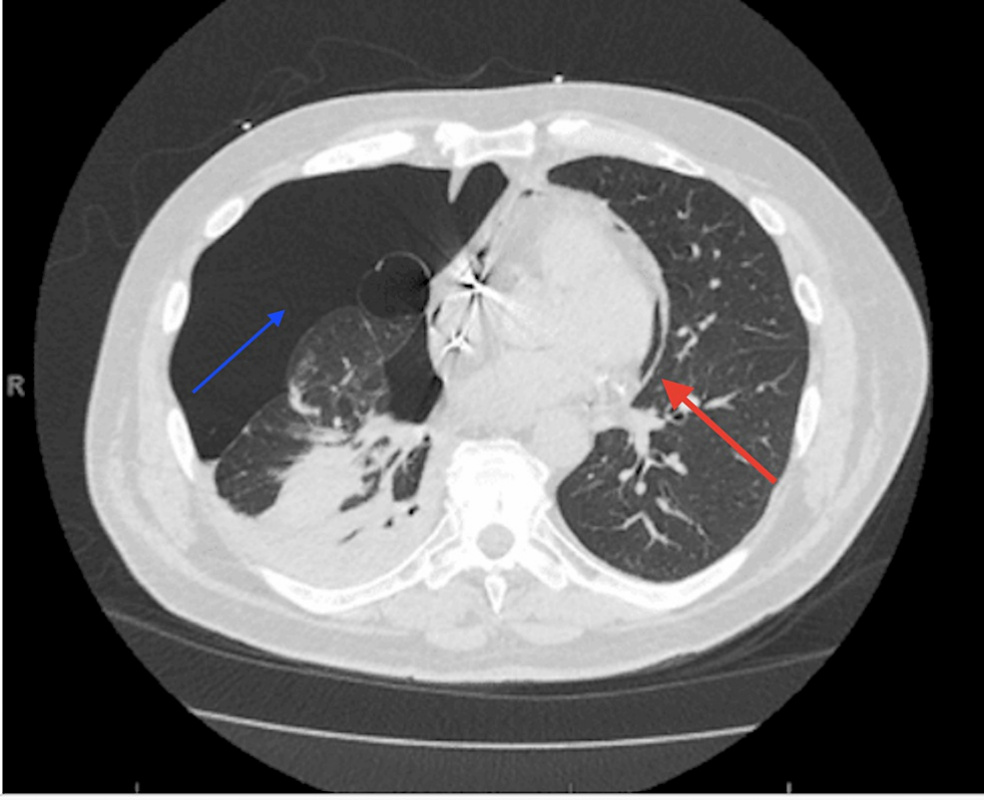
CT of the chest showing both a right-sided pneumothorax (blue arrow) and a contralateral pneumopericardium indicated by the red arrow.

A 68-year-old male presented to the hospital with two syncopal events while driving, which was a new occurrence for the patient. The patient noted that during intermittent times of the day, he would experience dizziness, lightheadedness, and palpitations, resulting in a syncopal episode. The patient had normal heart sounds on examination, bradycardic, with no gallops or rubs, and his lungs were clear to auscultation. The patient also had negative orthostatic vital signs. The patient had a past medical history of coronary artery disease status post percutaneous coronary intervention to the obtuse marginal artery and left circumflex, hypertension, and benign prostatic hyperplasia. The patient's coronary artery disease was managed with Aspirin, Plavix, and statin therapy. The patient had a completed 12-lead electrocardiogram (EKG) while admitted, which showed sinus bradycardia with long pauses of up to 6.2 seconds. Thus, the patient further underwent a dual chamber pacemaker implantation through the left subclavian vein approach, and using the modified Seldinger technique, a 27 F introducer was placed into the left subclavian vein. Through this introducer, the atrial and ventricular lead was advanced to the high right atrium and right ventricular apex where excellent pacing and sensing threshold was observed. It was noted that there were multiple attempts made to place the right atrium lead. After the procedure, the patient developed severe right-sided chest pain, and a repeat 12-lead EKG showed diffuse ST segment elevations, consistent with pericarditis. An immediate chest X-ray was obtained which showed a right-sided pneumothorax (Figure 1), and a chest CT showed a large right-sided pneumothorax and a contralateral pneumopericardium (Figures 2, 3). The patient had a chest tube placed and his pacemaker was interrogated and showed stable function. The patient also had a repeat CT chest which showed significant improvement in the pneumopericardium, and pneumothorax and he was treated conservatively.

After the patient’s hospitalization, he continued to improve after his chest tube was removed and he followed up with cardiology outpatient and remained stable. The patient also completed repeat chest imaging, which showed resolution of the patient’s pneumothorax and pneumopericardium. He denied any further episodes of chest pain, shortness of breath or syncopal episodes and his pacemaker continued to remain stable and functional on subsequent interrogation.

## Discussion

Ipsilateral pneumothorax and cardiac perforation are not uncommon following pacemaker implantation; however, contralateral pneumothorax and pneumopericardium are extremely rare, with currently only three previous case reports documented [1,2]. In our patient, it appears that the right atrium lead perforated through the right atrium into the right pleural space creating a fistula between the pericardial and pleural spaces, seen in Figure 2 above. This resulted in pneumothorax and a contralateral pneumopericardium. Interestingly, previous case reports, such as Nantsupawat et al. [4], had demonstrated a very large P-wave on one of the perforations with symptoms developing one day after the procedure and all perforations had occurred with active fixation leads from three separate manufacturers [1,2]. In our patient, there was excellent intrinsic sensing of the P-waves, and no lead extraction was required.

Signs and symptoms in these patients include pleuritic chest pain, shortness of breath, hypotension, or completely asymptomatic. The first step usually taken is a lead interrogation and chest radiography is the mainstay used to detect a pneumothorax [3,4]. However, in our case, the CT scan revealed the pneumothorax and contralateral pneumopericardium. Risk factors for perforation include variations in the right atrium structure, the number of attempts in placing the lead, and the lead design [1,5,6]. In our patient, there were three attempts at lead placement.

Due to the rarity of pneumothorax and contralateral pneumopericardium after pacemaker implantation, there’s not a well-established management protocol [3,4,6]. Some patients may be treated with both a chest tube insertion and a lead extraction; however, conservative management may also be pursued [1,2,6]. Some patients may require a chest tube depending on the amount of air or blood leakage and the hemodynamic status of the patient [1]. If a pneumothorax involves greater than 10% of the pleura and if there was an associated hemopneumothorax, a chest tube should be inserted [1,2,6].

## Conclusions

Our case demonstrated a successful outcome with conservative management, after a patient developed a pneumothorax and contralateral pneumopericardium after a dual chamber pacemaker insertion. Conservative treatment may be used as a treatment option depending on the patient’s hemodynamic status, lead status and size of pneumothorax and pneumopericardium. However, the ideal management protocol for these patients would require further study.
